# Chronic early life lead (Pb^2+^) exposure alters presynaptic vesicle pools in hippocampal synapses

**DOI:** 10.1186/s40360-016-0098-1

**Published:** 2016-11-02

**Authors:** Sara Rose Guariglia, Kirstie H. Stansfield, Jennifer McGlothan, Tomas R. Guilarte

**Affiliations:** Department of Environmental Health Sciences, Mailman School of Public Health, Columbia University, 722 West 168th Street, New York, NY 10032 USA

**Keywords:** Lead (Pb^2+^), Synapses, Presynaptic, Vesicles, Postsynaptic Density (PSD), Mitochondria

## Abstract

**Background:**

Lead (Pb^2+^) exposure has been shown to impair presynaptic neurotransmitter release in both in vivo and in vitro model systems. The mechanism by which Pb^2+^ impairs neurotransmitter release has not been fully elucidated. In previous work, we have shown that Pb^2+^ exposure inhibits vesicular release and reduces the number of fast-releasing sites in cultured hippocampal neurons. We have also shown that Pb^2+^ exposure inhibits vesicular release and alters the distribution of presynaptic vesicles in Shaffer Collateral – CA1 synapses of rodents chronically exposed to Pb^2+^ during development.

**Methods:**

In the present study, we used transmission electron microscopy to examine presynaptic vesicle pools in Mossy Fiber-CA3 synapses and in Perforant Path-Dentate Gyrus synapses of rats to determine if in vivo Pb^2+^ exposure altered presynaptic vesicle distribution in these hippocampal regions. Data were analyzed using *T*-test for each experimental endpoint.

**Results:**

We found that Pb^2+^ exposure significantly reduced the number of vesicles in the readily releasable pool and recycling pool in Mossy Fiber-CA3 terminals. In both Mossy Fiber-CA3 terminals and in Perforant Path-Dentate Gyrus terminals, Pb^2+^ exposure significantly increased vesicle nearest neighbor distance in all vesicular pools (Rapidly Releasable, Recycling and Resting). We also found a reduction in the size of the postsynaptic densities of CA3 dendrites in the Pb^2+^ exposed group.

**Conclusions:**

In our previous work, we have demonstrated that Pb^2+^ exposure impairs vesicular release in Shaffer Collateral - CA1 terminals of the hippocampus and that the number of docked vesicles in the presynaptic active zone was reduced. Our current data shows that Pb^2+^ exposure reduces the number of vesicles that are in proximity to release sites in Mossy Fiber- CA3 terminals. Furthermore, Pb^2+^ exposure causes presynaptic vesicles to be further from one another, in both Mossy Fiber- CA3 terminals and in Perforant Pathway – Dentate Gyrus terminals, which may interfere with vesicle movement and release. Our findings provide a novel in vivo mechanism by which Pb^2+^ exposure impairs vesicle dynamics and release in the hippocampus.

## Background

Effective neurotransmission requires appropriate expression, packaging, release, reception and degradation or reuptake of neurotransmitters [1]. Mechanisms underlying the release of neurotransmitters are highly complex and require precise interactions between pre-synaptic membrane proteins, vesicular proteins, ions and energy [2]. Exposure to lead (Pb^2+^) has been shown to inhibit the release of neurotransmitters, including glutamate and γ-aminobutyric acid (GABA), in various in vivo, in vitro and ex vivo models [3–6].

It is well known that the brain is a primary target of Pb^2+^ toxicity [7, 8]. Pb^2+^ can readily cross the blood brain barrier (BBB), and is found in brain homogenate following Pb^2+^ exposure [9], which is likely due to the ability of Pb^2+^ to substitute for Ca^2+^ ions [10, 11]. On the cellular level, Pb^2+^ exposure results in a myriad of direct effects in brain, which include apoptosis, excitotoxicity and alterations in neurotransmitter storage and release [12–20].

The mechanism by which Pb^2+^ exposure impairs vesicular release appears to involve presynaptic releasing sites [18–20]. In hippocampal neuron cultures, we have shown that Pb^2+^ exposure increased the number of nascent presynaptic contact sites. These release sites may be immature and lack the necessary vesicular release machinery proteins, thus contributing to reduced vesicular docking and release [18]. The mechanism by which Pb^2+^ exposure decreases the number of vesicular docking sites may involve retrograde Brain Derived Neurotrophic Factor (BDNF) – Receptor Tyrosine Kinase B (TrkB) receptor signaling [19]. Pb^2+^ is a potent inhibitor of the N-Methyl-d-Aspartate (NMDA) receptor, [21–23] whose activation is essential for cAMP Response Element (CREB) phosphorylation and subsequent BDNF expression [19]. Inhibition of the NMDA receptor by Pb^2+^ exposure reduces the expression of BDNF and impairs presynaptic BDNF-TrkB receptor signaling [5, 18, 19]. Reduced BDNF-TrkB interaction on presynaptic sites decreases the phosphorylation of synapsin-1, a vesicular protein which is important in vesicle-synaptic membrane interactions and is imperative to vesicular release [23–25]. Previously, we have shown that synapsin I phosphorylation at sites 4 (serine 62) and 5 (serine 67) were significantly decreased by Pb^2+^ exposure with no effect on total synapsin I protein levels [18]. The effect of Pb^2+^ on these particular signaling mechanisms is not reversed when Pb^2+^ is removed from the system which suggests that Pb^2+^ exposure results in persistent, deleterious effects on neurotransmission. On the other hand, the inhibitory effect of Pb^2+^ on L-type calcium channels can be reversed with removal of Pb^2+^ [26]. Therefore, the permanent effect of Pb^2+^ exposure on vesicular exocytotic mechanisms appears to be exceedingly important in Pb^2+^ exposure induced deficits in neurotransmission.

Mechanistic studies demonstrate that Pb^2+^ can have an effect on multiple cellular constituents that are involved in neurotransmission, thus leading to differential effects of Pb^2+^ exposure on neurotransmission in different brain regions, even within the same structure. For example, chronic, low-level exposure to lead has been shown to reduce long term potentiation (LTP) in NMDA receptor-dependent CA1 synapses, while having no effect on LTP in NMDA receptor - independent synapses of the CA3 region of the hippocampus [27]. Recently, we have shown that Pb^2+^ markedly inhibits presynaptic vesicular release in the hippocampal Shaffer Collateral – CA1 synapses in young adult rats. In support of previous data, impairments in vesicular release were found in CA1 but not in CA3 immediately following stimulation. This decrease in vesicular release in CA1 was found along with a decrease in both the rapidly releasable pool/docked (RRP/docked) vesicle pools as well as the recycling pool of vesicles, with no overall reduction in the total number of presynaptic vesicles. Furthermore, vesicles in the Pb^2+^ exposure group were further apart from one another, irrespective of the distance of the vesicle to the presynaptic active zone (PAZ). Interestingly, at later time points, impairments in vesicular release became apparent in the CA3, demonstrating the differential effects of Pb^2+^ on neurotransmission in the CA1 and CA3 brain regions [20].

Mitochondria are organelles that are intricately involved in neurotransmission, as they provide energy for vesicular biogenesis, packaging, movement and release [28]. Mitochondria are typically synthesized in the cell body, are sent to the axonal terminal for energy and then sent back to the cell body for degradation [29]. Exposure to Pb^2+^ has been shown to have numerous detrimental effects on mitochondria [30–32]. Our previous work has shown that chronic Pb^2+^ exposure reduces the number of mitochondria found in Shaffer Collateral- CA1 synapses [20]. Mechanistically, Pb^2+^ exposure can impair ATP synthesis, which would in turn limit energy-expending activities. Therefore, the effect of Pb^2+^ on presynaptic mitochondria may contribute to impaired vesicular release.

In light of our previous work, which demonstrated that there was a latent but significantly impaired neurotransmission in the CA3 following Pb^2+^ exposure, we sought to determine if Pb^2+^ exposure could affect the distribution of vesicular pools in the presynaptic terminals of asymmetric Mossy Fiber-CA3 synapses, using electron microscopy. We also examined the effect of Pb^2+^ exposure on asymmetric Perforant Pathway-Dentate Gyrus synapses, another region of the hippocampus in which Pb^2+^ exposure has been shown to impair long term potentiation [33]. Asymmetric synapses can be identified using electron microscopy by identification of the postsynaptic density (PSD). We have selected to examine the synapses of rats exposed to 1500 ppm lead. Feeding rats this concentration of lead yields a blood lead level of approximately 21 ug/dL. Previous studies have shown that a BLL of approximately 27 ug/dL yields a significant change in NMDA receptor expression as compared to low level Pb^2+^ exposure and highly concentrated Pb^2+^ exposure [9]. Since we examined asymmetric synapses via identification of the PSD, which typically contain NMDA receptors, we thought that a creating a model with a BLL comparable to that which produces Pb^2+^ mediated effects on NMDA receptor would allow us to characterize the maximal effect of Pb^2+^ on vesicular pools. Furthermore, we have found that this particular exposure produced profound spatial learning deficits [6, 34], which likely reflect the effect of this particular concentration of Pb^2+^ on hippocampal function. Additionally, we studied the number and size of mitochondria in the presynaptic terminals to determine if there were changes in mitochondrial size, number and distribution that could affect energy availability in the presynaptic terminals and examined the size of the postsynaptic density in asymmetrical synapses.

## Methods

### Chemicals

All chemicals used for electron microscopy (Glutaraldehyde, Paraformaldehyde, Osmium Tetroxide, Uranyl Acetate, Ethanol, Propylene Oxide and Spurr’s Low Viscosity Resin Embedding Kit) were purchased from Electron Microscopy Sciences (Hatfield, PA, USA).

### Animals

Adult female Long-Evans rats were purchased from Charles River, Inc. (Wilmington, MA) and fed 0 (control) or 1500 ppm lead acetate (PbAc) in the diet (Dyets, Bethlehem, PA) 10 days prior to breeding with normal Long-Evans males. Litters were culled to 10 on postnatal day 1 (PN1). Dams were maintained on their respective diet until weaning of pups. After weaning, offspring remained on respective maternal diet until PN 50. Rats were housed in pairs in rectangular plastic cages at 22 ± 2 °C on a 12/12 light:dark cycle. Food and water were allowed *ad libitum*. Rats were provided with corn cob bedding which was changed on a weekly basis. 10 litters of rats were bred for these studies. We used a litter based design in which one male rat per litter was randomly selected for inclusion into either the control group or the control group (*n* = 10, *n* = 5 Pb^2+^; *n* = 5 control). The number of animals to be used was calculated with a Power Analysis using G*Power 3.1 statistical software. This study was carried out in strict accordance with the recommendations in the Guide for the Care and Use of Laboratory Animals of the National Institutes of Health. The protocol was approved by Columbia University Institutional Animal Care and Use Committees (AC-AAAF4810). All non-survival procedures were performed under sodium pentobarbital anesthesia, and all efforts were made to minimize suffering.

### Specimen preparation

All perfusion procedures were carried out during the light cycle (1300–1600 h). At PN 50, Long-Evans male rats were anesthetized with 20 mg/kg pentobarbital via intraperitoneal injection in their home cage. Pentobarbital was selected because it acts upon the GABAA receptor to induce anesthesia. Blood was transcardially perfused with 2.5 % Glutaraldehyde + 2 % Paraformaldehyde in 0.1 M Phosphate Buffered Saline (PBS). The brain was removed and post-fixed in the same solution overnight at room temperature (RT). Brains were sectioned into 500 um slices with a vibratome. Sections were laid flat and two regions of interest (Perforant Pathway – Dentate Gyrus synapses and Mossy Fiber – CA3 synapses) were dissected from the right hippocampus using a hole-punch method (Fig. 2). The right side of the brain is typically associated with visuospatial processing and spatial memory [35]. Since Pb^2+^ exposure results in decreased spatial learning ability, we chose to investigate the right hippocampus because it isassociated with spatial learning [6, 34]. Male rats, in particular, exhibit prominent laterality [36]. Dissected tissue was placed in the 2.5 % Glutaraldehyde + 2 % Paraformaldehyde in PBS mixture for 3 additional h at RT and rinsed with PBS. Secondary fixation in 1 % osmium tetraoxide in PBS was then done for 60 m at RT. Following osmium fixation, tissue was rinsed in PBS then rinsed in water to remove all traces of phosphate from samples. Tissue was subsequently dehydrated in 50 % ethanol, a mixture of 70 % ethanol + 1 % uranyl acetate, 85 % ethanol and 2 changes of 100 % ethanol (15 m per step). Tissue was then placed in the transition solvent propylene oxide twice (15 m per step) and was left to infiltrate in a 1:1 mixture of propylene oxide-Spurr’s Resin overnight at RT. Steps involving osmium tetraoxide and uranyl acetate were done in containers covered with foil to block light. Tissue was transferred to pure Spurr’s Resin for infiltration for 24 h at RT. Tissue was then placed into Beem Capsules with fresh Spurr’s Resin, allowed to sit for 30 m and then placed in a 70 C oven for 24 h for polymerization. After polymerization, ultrathin sections (70 nm) were obtained using a Leica Ultracut ultramicrotome and placed onto 200 mesh copper grids. 2 um of tissue was cut in between each collected section to prevent repeat analysis of any synapses. Sections on grids were then stained with uranyl acetate for 45 m, rinsed with water, stained with lead citrate for 90 s, rinsed with water and left to dry on clean filter paper.

### Imaging

**Fig. 1:**
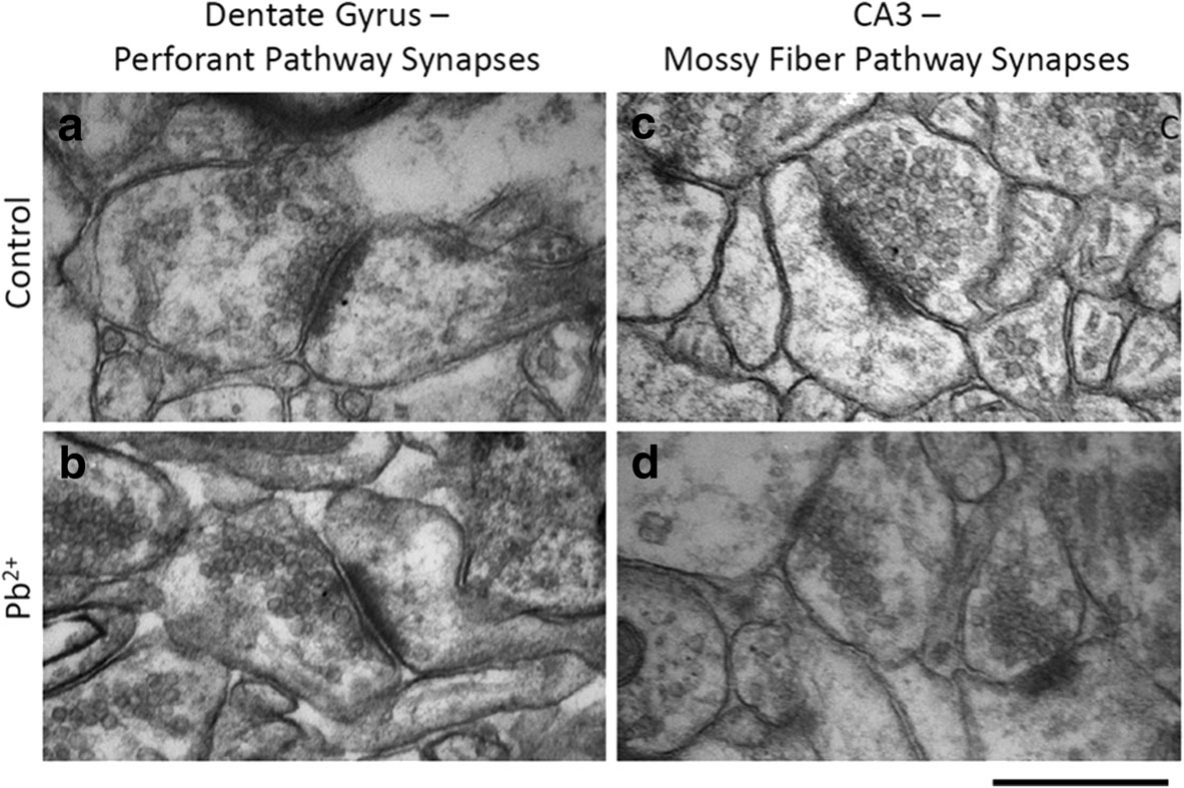
Dentate Gyrus –Perforant Pathway (DG-PP) synapses and CA3-Mossy Fiber Pathway (CA3-MF) Synapses. (**a**) Control DG-PP synapse, (**b**) Pb^2+^ DG-PP synapse, (**c**) Control CA3-MF synapse, and (**d**) Pb^2+^ CA3-MF synapse. There are more docked vesicles in presynaptic terminals of control rats than there are in Pb^2+^ exposed rats. Docked vesicles are those that are physically contacting the presynaptic active zone (PAZ). There is also a reduction in recycling pool vesicles in Pb^2+^ exposed groups. No overall reduction in total vesicle number was found. The postsynaptic density was markedly smaller in the CA3-MF terminals of Pb^2+^ exposure groups. Scale bar = 500 nm

Tissue was examined under a Hitachi 7500 Transmission Electron Microscope operated at 80 kV. Images were obtained at 100,000x magnification using an AMT digital camera and software. For each hippocampus under investigation (10 total; 5 Control and 5 Pb^2+^), a total of 80 images of simple, asymmetric synapses were obtained (*n* = 40 for Mossy Fiber - CA3 and *n* = 40 for Perforant Pathway - DG synapses; Fig. 1a-d). 5 synapses were imaged from each grid. Synapses were spaced by a minimum of one grid box to reduce bias. The microscopist was blinded to experimental groups.

### Image analysis

**Fig. 2:**
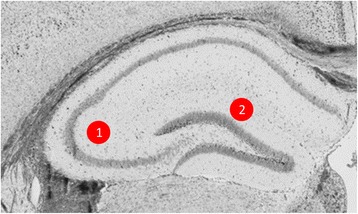
Regions that were sampled using for electron microscopy analysis. A hole-punch method was used to dissect (1) CA3- Mossy Fiber synapses and (2) Perforant Pathway – Dentate Gyrus synapses

A total of 34 variables per animal were measured, comprising 17 variables from two independent hippocampal brain regions (Mossy Fiber - CA3 and Perforant Pathway - DG). The presynaptic active zone (PAZ) and the center of each pre-synaptic vesicle was marked using ImageTool. The distance between each vesicle and the PAZ as well as the distance between each vesicle and its nearest neighbor was calculated using ImageTool coordinates in LoClust [37]. The area of each axon terminal was measured as well as the diameter of each vesicle using ImageJ. The PAZ length was also measured using ImageJ. PAZ membrane appears more electron dense after staining than surrounding membranes, which allows for measurement. The postsynaptic density (PSD) length was measured using ImageJ. The PSD is large and electron dense after staining, which facilitates measurement. Vesicles were classified as RRP/docked if they were physically contacting the PAZ. Vesicles were classified as belonging to the recycling pool if their center was within 200 nm of the PAZ. Vesicles were considered part of the reserve pool if their vesicular center was greater than 200 nm from the active zone. These criteria have been established by other morphological and molecular studies of vesicular populations [38, 39]. The number and diameter of mitochondria in the pre-synaptic terminal was also determined (Fig. 2a-c).

### Statistics

To obtain the appropriate number of images for these analyses, we calculated the number of images needed using G*Power statistical software. 40 images of the Mossy Fiber - CA3 synapses were needed for each rat. We then made 17 measurements per image. Each measurement reflected a single experimental endpoint under investigation (ex. Docked vesicle number, PSD length, number of mitochondria in terminals, etc). We then used the 40 images to obtain the mean measurement for each of the experimental endpoints per rat. This led to the generation of 10 mean values for each experimental endpoint under investigation (ex. 5 Control means vs. 5 Pb^2+^ means for the Mossy Fiber - CA3 synapses per endpoint). We used a *T*-test to compare the mean values of the Control vs. Pb^2+^ exposed groups to determine if there were differences in the particular endpoint being investigated (GraphPad Prism). Data is reported as SEM, as we were examining differences between the means of each experimental group [40]. We also analyzed the same endpoints in the Perforant Pathway - DG synapses, a functionally distinct part of the hippocampus, using the same strategy. Differences were considered significant if *p* < 0.05.

## Results

### Blood Lead Level (BLL) and weight

The Pb^2+^ exposure paradigm used in the present study does not produce any overt toxicity based on body weight gain. Body weight at postnatal day 50 (PN50) were: 294.4 ± 4.8 grams (*n* = 24) for control animals and 281.6 ± 6.9 grams for Pb^2+^ -exposed animals. Blood Pb^2+^ levels of littermates to animals used in this study at PN50 were: 0.8 ± 0.3 μg/dL (*n* = 11) for control animals and 21.1 ± 1.6 μg/dL (*n* = 15) for Pb^2+^-exposed animals.

### Analysis of vesicle number

There were marked changes in the presynaptic vesicular pools of rats that were exposed to Pb^2+^ (Fig. 1). Chronic Pb^2+^ exposure resulted in a significant reduction in the number of RRP/docked vesicles in Mossy Fiber terminals (*p = 0.0236;* Table 1 *, IA).* Exposure to Pb^2+^ also resulted in a near-significant reduction in the number of vesicles in the RRP/docked vesicles in Perforant Path terminals (*p = 0.0989;* Table 1 *, IA)*. There was a significant reduction in the number of recycling pool vesicles in Mossy Fiber terminals (*0.0187;* Table 1 *, IB*) in rats exposed to Pb^2+^. The total number of vesicles found in terminals of control and Pb^2+^ treated rats were similar in both the Mossy Fiber (*p = 0.4936;* Table 1 *, ID)* and Perforant Pathway terminals (*p = 0.4603;* Table 1 *, ID*). In general, the diameter of vesicles in the RRP/docked vesicle pool, the recycling pool, the resting pool, and all vesicle pools combined in Mossy Fiber and Perforant Path terminals were not changed by Pb^2+^ exposure (Table 1, IIA-D).

**Table 1:** Vesicle, clustering, and mitochondrial measurements in Dentate Gyrus –Perforant Pathway (DG-PP) synapses and CA3-Mossy Fiber Pathway (CA3-MF) Synapses

	CA3 – Mossy Fiber Terminals			Dentate Gyrus – Perforant Pathway Terminals		
	Control (Mean ± SEM)	Pb^2+^ (Mean ± SEM)	*P* value	Control (Mean ± SEM)	Pb^2+^ (Mean ± SEM)	*P* value
I. Raw Vesicle Counts						
A) Rapidly Releasable/Docked Vesicle Pool	8.527 ± 0.8456	6.018 ± 0.001	0.0236	7.013 ± 1.003	5.232 ± 0.9928	0.0989
B) Recycling Vesicle Pool	35.19 ± 7.804	29.26 ± 7.381	0.0187	31.48 ± 9.315	26.09 ± 5.835	0.1814
C) Resting Vesicle Pool	43.64 ± 5.570	51.80 ± 7.427	0.2271	51.66 ± 8.374	59.80 ± 11.44	0.2586
D) Total Number of Vesicles	87.36 ± 10.61	87.17 ± 1.761	0.4936	90.07 ± 9.363	91.12 ± 10.46	0.4603
II. Vesicular Diameters						
A) Rapidly Releasable/Docked Diameter	20.67 ± 1.644	21.20 ± 1.137	0.3995	20.82 ± 1.521	23.27 ± 2.12	0.3894
B) Recycling Vesicle Diameter	22.09 ± 0.8383	22.77 ± 0.6942	0.2799	22.10 ± 0.8234	24.41 ± 2.072	0.1695
C) Resting Vesicle Diameter	24.83 ± 1.746	24.70 ± 1.322	0.4764	24.69 ± 2.481	26.28 ± 2.506	0.3343
D) Average Diameter of all vesicles	23.77 ± 1.406	23.84 ± 0.8775	0.4843	23.72 ± 1.565	25.64 ± 2.094	0.2449
III. Length Measurements						
A) PSD length	309.1 ± 13.48	259.7 ± 18.74	0.0382	269.3 ± 9.680	266.6 ± 15.31	0.4432
B) PAZ length	311.6 ± 11.15	281.2 ± 19.53	0.0899	265.6 ± 14.45	308.2 ± 26.90	0.1553
IV. Nearest Neighbor Distance (Clustering)						
A) Rapidly Releasable/Docked + Recycling (0–200 nm)	40.19 ± 1.398	45.56 ± 1.498	0.0062	41.75 ± 0.7873	48.57 ± 1.115	0.0001
B) Resting (201–500 nm)	42.04 ± 1.834	48.47 ± 1.157	0.0020	45.35 ± 0.7979	51.19 ± 0.7168	0.0001
V. Mitochondrial Measurements						
A) Terminals with mitochondria	15.40 ± 2.227	17.60 ± 1.806	0.2340	27.00 ± 3.050	22.60 ± 3.280	0.1793
B) Terminals with multiple mitochondria	2.00 ± 0.5477	1.250 ± 0.2500	0.1316	4.200 ± 0.9695	2.600 ± 0.5099	0.0970
C) Total number of mitochondria	48.60 ± 6.577	54.20 ± 7.690	0.5972	69.60 ± 0.4964	62.80 ± 0.2518	0.1375
D) Diameter of mitochondria	231.6 ± 16.18	224.4 ± 15.53	0.7566	221.8 ± 0.8072	226.5 ± 8.529	0.3500
E) Large mitochondria (300 nm or greater)	4.800 ± 1.594	6.800 ± 0.9695	0.1624	8.00 ± 2.345	7.200 ± 1.530	0.3924

We also measured postsynaptic density (PSD) and presynaptic active zone (PAZ) length in Mossy Fiber-CA3 and Perforant Path-Dentate Gyrus synapses. We found that PSD length in CA3 dendrites was significantly reduced in rats chronically exposed to Pb^2+^ (*p = 0.0382;* Table 1 *, IIIA*). There was no change in Dentate Gyrus dendrites (*p = 0.4432;* Table 1 *, IIIA)*. PAZ length was similar in the dendridic fields of the control and Pb^2+^ exposed rats (*p = 0.0899 and p = 0.1553, respectively;* Table 1 *, IIIB*). The reduction in the RRP/docked vesicle pool may contribute to the reduced size of the PSD that was found in the striatum radiatum of CA3 dendrites.

### Vesicle clustering (nearest neighbor distance)

A reduction in vesicle clustering was the most ubiquitous effect of chronic Pb^2+^ exposure. Vesicles were more dispersed in Pb^2+^ exposed animals relative to controls in both Mossy Fiber and Perforant Pathway terminals. For example, the nearest neighbor distance in vesicles that are within 200 nm of the PAZ in both the Mossy Fiber and the Perforant Path were significantly greater in Pb^2+^ exposed animals as compared to controls (*p = 0.0062 and 0.0001, respectively;* Table 1 *, IVA).* Similarly, nearest neighbor distance of vesicles in the resting pool (greater than 200 nm from the PAZ) are also less clustered in Mossy Fiber and Perforant Pathway axon terminals from Pb^2+^ exposed animals as compared to controls (*p = 0.0020 and 0.0001, respectively;* Table 1 *, IVB).*

### Mitochondria

Chronic Pb^2+^ exposure did not result in any remarkable changes in mitochondria number or size. In the Pb^2+^ exposure group, there are no differences in the total number of terminals with mitochondria (*p = 0.2340 and p = 0.1793, respectively,* Table 1 *, VA)*. In Pb^2+^ exposed rats, there is a modest reduction in Perforant Pathway terminals that contained multiple mitochondria, but the difference did not reach statistical significance (*p = 0.0970, respectively;* Table 1 *, VB)*. The mean number of total mitochondria in both Mossy Fiber and Perforant Pathway terminals were not significantly different from controls (*p = 0.5972 and p = 0.1375, respectively;* Table 1 *, VC).* The mean diameter of Mossy Fiber and Perforant Pathway terminal mitochondria was similar between control and Pb^2+^ exposed groups (*p = 0.7566 and p = 0.3500, respectively,* Table 1 *, VD*). The number of mitochondria with a cross sectional diameter greater than 300 nm were not significantly between Pb^2+^ exposed groups and control groups in Mossy Fiber terminals or in Perforant Pathway Terminals (*p = 0.1624 and p = 0.3924, respectively;* Table 1 *, VE).*

## Discussion

The purpose of our work was to determine if Pb^2+^ effected the distribution of presynaptic vesicular pools, distribution of vesicles, and mitochondrial size in the Mossy Fiber – CA3 and Perforant Pathway – Dentate Gyrus terminals of the hippocampus of rats. Our work shows that Pb^2+^ exposure results in a decreased number of RRP/docked vesicles and recycling pool vesicles in Mossy Fiber – CA3 terminals. Pb^2+^ exposure did not alter vesicle number in the different pools of the Perforant Pathway – Dentate Gyrus terminals. Pb^2+^ treatment did not appear to affect the size of the vesicles or affect the biogenesis of the vesicles, as there were no differences in the total number of vesicles present in the terminals. Nearest neighbor distance of vesicles in both the Mossy Fiber – CA3 terminals and in Perforant Pathway – Dentate Gyrus terminals of Pb^2+^ exposed animals were significantly greater than controls, indicating that the vesicles were more dispersed in the Pb^2+^ exposed animals.

With respect to the RRP/docked vesicles, Pb^2+^ exposure consistently induced a significant reduction in the number of vesicles that were contacting the PAZ in Mossy Fiber terminals. In primary hippocampal culture, we found that Pb^2+^ exposure increased the number of nascent presynaptic docking sites, but many of these docking sites were lacking NSF attachment protein receptor complex, which is involved in vesicular exocytosis [5, 7]. It seems plausible that Pb^2+^ exposure may have a similar effect *in vivo,* which may contribute to the reduced number of RRP/docked vesicles that were found in the Pb^2+^-exposed animals. In our previous work, we found that Pb^2+^ exposure reduced the expression of synaptophysin and synaptobrevin, two vesicular proteins that are imperative to vesicular docking and release [5], which may contribute to the Pb^2+^ -induced changes that we have found in our current study.

We found fewer vesicles in the recycling pool of Pb^2+^ exposed animals, but no decrease in the total number of vesicles in each terminal. Pb^2+^ exposure also altered the distribution of the vesicles, as they were significantly further apart from one another in all hippocampal regions that were examined. Our data suggests that Pb^2+^ does not affect the biogenesis of the vesicles. The spatial arrangement of vesicles relative to the PAZ was highly affected by Pb^2+^ exposure, which likely reflects Pb^2+^ induced changes to the expression and activation of vesicular trafficking proteins and scaffolding proteins, such as synapsin. We have previously demonstrated that Pb^2+^ reduces phosphorylation of synapsin I in primary hippocampal culture. Synapsin I is a vesicular protein that in its unphosphorylated state keeps reserve pool vesicles bound to actin filaments. Once phosphorylated, vesicles are released from actin filaments and allowed to move into the recycling and RRP/docked vesicle pools [23–25]. We have shown that synapsin I phosphorylation at sites 4 (serine 62) and 5 (serine 67) were significantly decreased by Pb^2+^ exposure with no effect on total synapsin I protein levels [18]. It is possible that Pb^2+^ reduces synapsin I phosphorylation in vivo as well, which may contribute to the decreased number of vesicles in the recycling and RRP/docked vesicle pools that we observed in the present study.

Mitochondria, which provide energy for vesicular biogenesis, were largely unaffected by Pb^2+^ exposure in the Mossy Fiber-CA3 and Perforant Path- Dentate Gyrus synapses. This suggests that Pb^2+^ exposure may not effect energy production in these terminal regions, which is supported by the idea that there does not appear to be any reduction in vesicular biogenesis, evidenced by no observable changes in presynaptic vesicle number or diameter. Interestingly, we did find that Pb^2+^ exposure did reduce the number of Shaffer-Collateral terminals with multiple mitochondria [20], demonstrating varying aberrant pathological effects in the different hippocampal regions. This study does not rule out the possibility of mitochondrial dysfunction, but from the measures that we investigated, there appear to be no gross ultrastructural differences. Mitochondrial cristae morphology may reveal differences in energy production and may be examined in future investigations.

The PSD length in CA3 dendrites was significantly reduced in Pb^2+^ exposed animals. Pb^2+^ exposure may impair the maturation of the PSD. PSD-95 expression enhances pre-synaptic maturation [41] thus a Pb^2+^ exposure-induced reduction of PSD size may contribute to the presynaptic changes that we observed. In previous work, we found that Pb^2+^ exposure results in a significant reduction in Timm’s-positive staining in Mossy Fibers terminal fields [42]. Perhaps a reduction in the PSD in CA3 results in fewer axonal terminals that innervate this region. The length of the PSD in the dendrites of the Dentate Gyrus were not affected by Pb^2+^ exposure.

## Conclusions

Chronic, early life Pb^2+^ exposure alters the distribution of vesicles in both Mossy Fiber and Perforant Pathway terminals, which is in concurrence with our previous in vitro data. Such a redistribution of vesicles impairs fast and effective neurotransmission, which likely contributes to learning and memory impairments found in models of Pb^2+^ intoxication. The mechanisms underlying this redistribution of vesicles likely involve reduced expression in presynaptic proteins, such synaptophysin and synaptobrevin as well as reduced phosphorylation of synapsin I. The mechanisms underlying changes in the Mossy Fiber – CA3 and Perforant Pathway – Dentate Gyrus terminals likely involve different pathways, which reflect the physiological heterogeneity of these various hippocampal regions. Uncovering behavioral and pharmacological interventions that could help to rescue the distribution of vesicles will likely promote fast and efficient neurotransmission.

## Abbreviations

PAZ: Presynaptic active zone
PSD: Postsynaptic density
RRP: Readily releasable pool
